# Reduced HBV cccDNA and HBsAg in HBV-associated hepatocellular carcinoma tissues

**DOI:** 10.1007/s12032-018-1191-7

**Published:** 2018-08-16

**Authors:** Anchalee Tantiwetrueangdet, Ravat Panvichian, Pattana Sornmayura, Natthaporn Sueangoen, Surasak Leelaudomlipi

**Affiliations:** 1 Research Center, Faculty of Medicine, Ramathibodi Hospital, Mahidol University, Bangkok, Thailand; 2 Division of Medical Oncology, Department of Internal Medicine, Faculty of Medicine, Ramathibodi Hospital, Mahidol University, Rama 6 Road, Rajthevi, Bangkok, 10400 Thailand; 3 Department of Pathology, Faculty of Medicine, Ramathibodi Hospital, Mahidol University, Bangkok, Thailand; 4 Department of Surgery, Faculty of Medicine, Ramathibodi Hospital, Mahidol University, Bangkok, Thailand

**Keywords:** cccDNA, ddPCR, HBsAg, HBV, HCC

## Abstract

Approximately 50% of hepatocellular carcinoma (HCC) is attributable to chronic infection with hepatitis B virus (HBV). Serum hepatitis B surface antigen (HBsAg) is an important diagnostic marker of HBV infection, whereas intrahepatic HBV covalently closed circular DNA (cccDNA) is a surrogate marker of HBV persistence. This study aimed to investigate relationships between serum HBsAg, intrahepatic HBsAg, and intrahepatic cccDNA in HBV-associated HCC. Intrahepatic HBsAg was determined by immunohistochemistry in matched non-cancerous and HCC tissues from 88 patients; 56 patients (63.64%) were serum HBsAg positive. In serum HBsAg-positive group, intrahepatic HBsAg was positive staining in 73.2% of non-cancerous tissues, but only in 10.7% of HCC tissues. Significant correlation between serum HBsAg and intrahepatic HBsAg was observed in non-cancerous tissues (*p* < 0.001), but not in HCC tissues (*p* = 0.415). Absolute quantification of intrahepatic cccDNA was performed by droplet digital PCR in tissues from 30 patients; 18 patients (60%) were serum HBsAg positive. In serum HBsAg-positive group, intrahepatic cccDNA was detected in 66.66% of non-cancerous tissues, but only in 5.55% of HCC tissue; intrahepatic cccDNA levels in non-cancerous tissues were significantly higher than those in HCC tissues (*p* < 0.001), and correlated with serum HBsAg (*p* < 0.01). Significant correlations between intrahepatic HBsAg and intrahepatic cccDNA were found in both non-cancerous tissues (*p* < 0.01) and HCC tissues (*p* < 0.05). We concluded that HBV cccDNA and intrahepatic HBsAg in HBV-associated HCC tissues were significantly reduced, as compared with matched non-cancerous tissues. This warrants further investigation into the impacts and the cause(s) of cccDNA reduction in HBV-associated HCC tissues, which might yield novel immune-related therapy for HBV-associated HCC.

## Introduction

Hepatocellular carcinoma (HCC) is the fifth most common cancer in men and the ninth in women, as well as the second leading cause of cancer-related death globally [1, 2]. Almost 50% of all cases of HCC are associated with chronic infection with hepatitis B virus (HBV) [3, 4]. HBV belongs to a family of viruses known as Hepadnaviridae and encodes only four genes in a highly compact viral genome: the surface gene (S), the core gene (C), the X gene (X), and the polymerase gene (P) [5]. Hepatitis B surface antigen (HBsAg) is an important diagnostic marker of hepatitis B viral (HBV) infection. Previous studies have suggested that HBsAg may reflect the content of intrahepatic HBV covalently closed circular DNA (cccDNA), which has been proposed as a surrogate marker of HBV-infected hepatocytes [6, 7]. Several studies have investigated the HBV cccDNA level in HCC and matched non-cancerous tissues but the results are still inconclusive. Wong et al. [8] reported that in HBsAg-positive patients, HBV cccDNA level of tumor tissues was significantly higher than the level of non-tumor tissues. In contrast, two studies reported that HBV cccDNA level of cancer tissues was significantly lower than the level of non-cancerous tissues [9, 10]. In addition, Fu et al. [11] and Bai et al. [12] reported that there was no significant difference in intrahepatic HBV cccDNA levels between tumor and non-tumor liver tissues. Correlation between serum HBsAg levels and HBV cccDNA in tumor or non-neoplastic liver tissues of HBV-associated HCC patients was reported by Wang et al. [13]. In contrast, Wang et al. reported that serum HBsAg correlated poorly with intrahepatic HBV cccDNA in the tumor and non-tumor liver tissues [14]. Therefore, in this study, we investigated both HBsAg and HBV cccDNA in HCC and non-cancerous tissues so as to explore (1) the correlation between the serum HBsAg and the intrahepatic HBsAg; (2) the correlation between the serum HBsAg and the intrahepatic HBV cccDNA; and (3) the correlation between the intrahepatic HBsAg and the intrahepatic HBV cccDNA. Since HBV cccDNA is responsible for viral persistence, droplet digital PCR (ddPCR) technique, a highly sensitive PCR method, was applied for HBV cccDNA quantification in this study [15].

## Materials and methods

### Detection of HBsAg in HCC and matched non-cancerous tissues by IHC

Eighty-eight pairs of formalin-fixed paraffin-embedded (FFPE) tissues (HCC and matched non-cancerous tissues) were selected by an experienced pathologist. Serial 4-micron sections were cut and placed on positive-charged slides. These slides were deparaffinized in xylene and rehydrated through graded concentrations of ethanol and finally distilled water. Sections were then processed with an UltraVision LP Value Detection System (Lab Vision, USA). Briefly, sections were blocked with Hydrogen Peroxide Block for 15 min at room temperature, followed by Ultra V Block for 10 min at room temperature. Then, mouse monoclonal antibody to HBsAg (clone Ab-1, 1:200; Thermoscientific, USA) was applied to each sample for 1 h. Sections were incubated with Value Primary Antibody Enhancer for 30 min at room temperature; then, Value HRP Polymer was applied and the sections were incubated for 1 h at room temperature. DAB (3,3′-diaminobenzidine) was used as substrate to reveal the expression of each marker. Slides were counterstained with hematoxylin and mounted in permanent mounting medium. Tissues with omission of the specific antibody were used as negative controls. Slides were scanned with the Pannoramic MIDI digital slide scanner (3DHISTECH, Hungary). Positive staining of HBsAg was defined as cytoplasmic staining > 1 cell in each tissue section.

### Detection of HBV cccDNA by ddPCR

DNA of 30 paired HCC and matched non-cancerous of HCC tissues were extracted by QIAamp DNA Mini Kit (Qiagen, Germany). DNA concentration was measured by Qubit™ dsDNA BR Assay (Invitrogen, Oregon USA). To prepare the DNA template, 160 ng of DNA was cut with Exonuclease V (NEB, UK) in 10 µl reaction at 37 °C for 30 min, heat inactivated at 70 °C for 30 min. The ddPCR mixture consisted of 10 µl of 2X ddPCR Supermix for probe (Bio-Rad); 900 nM HBV cccDNA specific primers; 250 nM HBV cccDNA probe; and 1 µl DNA template (16 ng). The following primers and probe were used for amplification: 5′CTTCTCATCTGCCGGACC3′ (forward primer), 5′CACAGCTTGGAGGCTTGA3′ (reverse primer), and FAM-5′AGGCTGTAGGCATAAATTGGTCT-3′BHQ (probe) [15]. For each ddPCR reaction mixture, 70 µl droplet generation oil and 20 µl were added to the DG8 cartridge, then the droplets were produced by a droplet generator of the QX200 Droplet Digital PCR system (Bio-Rad). The droplets containing the PCR reaction mixture and droplet generation oil were then transferred to a 96-well PCR plate for amplification using the C1000 Touch Thermal Cycler (Bio-Rad) applying a thermalcycle beginning at 94 °C for 10 min, followed by 35 cycles of 94 °C for 30 s, 57 °C for 60 s, and a final cycle of 98 °C for 10 min. Subsequently, a droplet reader (Bio-Rad) was used to calculate the number of both positive and negative droplet events from each PCR reaction mixture. A PCR reaction mixture with no DNA template was used as a reference control for potential PCR contamination. Triplicate reactions were run for each sample. The ddPCR data were analyzed using the QuantaSoft analysis software (Bio-Rad), which calculates the total number of droplets (positive droplets plus negative droplets).

### Serum hepatitis B surface antigen (HBsAg) assay

Chemiluminescent microparticle immunoassays (CMIA) for the qualitative detection of hepatitis B surface antigen (HBsAg) in serum from the patients were performed using ARCHITECT HBsAg Qualitative II assay (Abbot Laboratories, Illinois, USA).

### Serum alpha-fetoprotein (AFP) assay

Electrochemiluminescence immunoassays (ECLIA) for the in vitro quantitative determination of alpha-fetoprotein (AFP) in serum from the patients were performed using the AFP kit with a cobas e601 analyzer (Roche Diagnostics Limited GmbH, Mannheim, Germany).

### Statistical analysis

Statistical analyses were performed with SPSS v.11.5 (SPSS Inc., Chicago, Illinois, USA) or GraphPad Prism 7 (version 7.03). Correlation was determined using Chi-square test. The *p* values less than 0.05 were considered statistically significant.

## Results

### Correlation between serum HBsAg and intrahepatic HBsAg

**Fig. 1:**
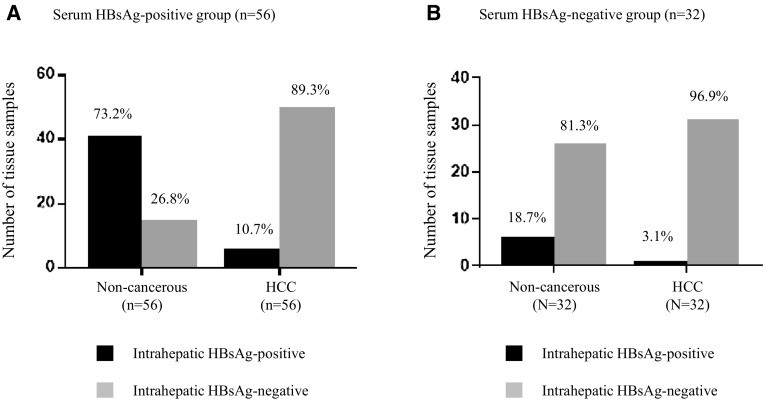
Intrahepatic HBsAg detected in non-cancerous and HCC tissues in serum HBsAg-positive group, *n* = 56 (**a**), and serum HBsAg-negative group, *n* = 32 (**b**)

Intrahepatic HBsAg was determined in 88 pairs of HCC and matched non-cancerous tissues by IHC. The data of serum HBsAg were collected from patient records. The clinicopathological features are illustrated in Table 1. Of 88 patients included in this study, serum HBsAg was determined as positive in 56 cases (63.64%) and negative in 32 cases (36.36%). In serum HBsAg-positive group, intrahepatic HBsAg was positive staining in 73.2% (41/56) of matched non-cancerous tissues but only in 10.7% (6/56) of HCC tissues, as shown in Fig. 1a. In serum HBsAg-negative group, intrahepatic HBsAg was positive staining in 18.7% (6/32) of matched non-cancerous tissues and only in 3.1% (1/32) of HCC tissues, as shown in Fig. 1b. The significant correlation between serum HBsAg and intrahepatic HBsAg in matched non-cancerous tissues was observed (*p* < 0.001) but there was no correlation between serum HBsAg and intrahepatic HBsAg in HCC tissues (*p* = 0.415), as shown in Table 2.

**Table 1:** Clinicopathological features of HCC patients whose intrahepatic HBsAg levels were measured by IHC

Patients (*n* = 88)	*n* (%)
Age (years)	
< 50	25 (28.4)
≥ 50	63 (71.6)
Sex	
Male	75 (85.23)
Female	13 (14.77)
Serum HBsAg	
Positive	56 (63.64)
Negative	32 (36.36)
Serum AFP	
< 500 ng/ml	62 (70.46)
≥ 500 ng/ml	24 (27.27)
Unknown	2 (2.27)
Tumor size	
< 5 cm	42 (47.73)
≥ 5 cm	46 (52.27)
TP53 expressions	
Negative	50 (56.82)
Positive	38 (43.18)
Ki-67 expression	
< 10%	45 (51.14)
≥ 10%	43 (48.86)

*HBsAg* hepatitis B surface antigen, *AFP* alpha-fetoprotein, *HCC* hepatocellular carcinoma, *TP53* tumor suppressor protein p53

**Table 2:** Correlation between serum HBsAg and intrahepatic HBsAg (*n* = 88) in matched non-cancerous tissues (A) and HCC tissues (B)

(A)	Intrahepatic HBsAg in matched non-cancerous tissues		
	Number positive (%)	Number negative (%)	
Serum HBsAg			
Positive	41 (73.2)	15 (26.8)	*p* < 0.0001
Negative	6 (18.7)	26 (81.3)	

(B)	Intrahepatic HBsAg in HCC tissues		
	Number positive (%)	Number negative (%)	
Serum HBsAg			
Positive	6 (10.7)	50 (89.3)	*p* = 0.415
Negative	1 (3.1)	31 (96.9)	

### Correlation between serum HBsAg and intrahepatic HBV cccDNA

**Fig. 2:**
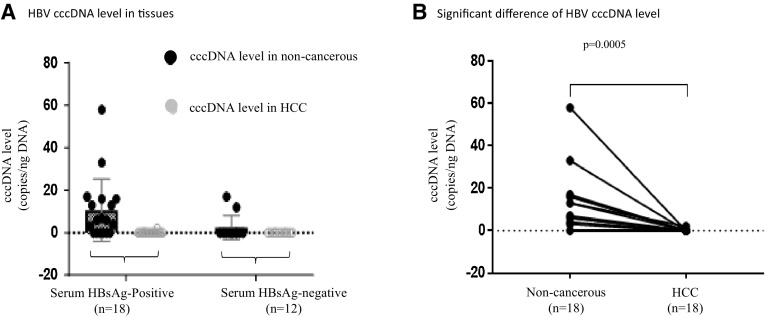
HBV cccDNA level detected by ddPCR in 30 pairs of matched non-cancerous and HCC tissues stratified by serum HBsAg, (**a**). Significant difference between cccDNA level in matched non-cancerous and HCC tissues in serum positive HBsAg group (*n* = 18) (*p* = 0.0005), (**b**)

Absolute quantification of intrahepatic cccDNA was performed by droplet PCR in 30 pairs of matched non-cancerous and HCC tissues from 30 patients, whose serum HBsAg was positive and negative in 60% (18/30) and 40% (12/30), respectively. The clinicopathological features are illustrated in Table 3. In serum HBsAg-positive group (18 samples), intrahepatic cccDNA was detected in 66.66% (12/18) of matched non-cancerous tissues (range 2–58 copies/ng DNA) but only in 5.55% (1/18) of HCC tissue (2 copies/ng DNA). In serum HBsAg-negative group (12 samples), intrahepatic cccDNA was detected in 16.66% (2/12) of matched non-cancerous tissues (12 and 17 copies/ng DNA) but not in all 12 HCC tissues, as shown in Table 4 and Fig. 2a. In serum HBsAg-positive group, cccDNA levels in matched non-cancerous tissues were significantly higher than in HCC tissues, *p* = 0.0005 (Fig. 2b). In addition, there was a significant correlation between serum HBsAg and cccDNA in matched non-cancerous tissues (*p* < 0.01).

**Table 3:** Clinicopathological features of HCC patients whose intrahepatic cccDNA levels were measured by ddPCR

Patients (*n* = 30)	*n* (%)
Age (years)	
< 50	13 (43.33)
≥ 50	17 (56.67)
Sex	
Male	26 (86.67)
Female	4 (13.33)
Serum HBsAg	
Positive	18 (60.0)
Negative	12 (40.0)
Serum AFP	
< 500 ng/ml	19 (63.33)
≥ 500 ng/ml	8 (26.67)
Unknown	3 (10.0)
Tumor size	
< 5 cm	12 (40.0)
≥ 5 cm	18 (60.0)
TP53 expressions	
Negative	16 (53.33)
Positive	14 (46.67)
Ki-67 expression	
< 10%	8 (26.67)
≥ 10%	22 (73.33)

*HBsAg* hepatitis B surface antigen, *AFP* alpha-fetoprotein, *HCC* hepatocellular carcinoma, *TP53* tumor suppressor protein p53

**Table 4:** Correlation between serum HBsAg and intrahepatic HBV cccDNA (*n* = 30) in matched non-cancerous tissues (A), and in HCC tissues (B)

(A)	HBV cccDNA in matched non-cancerous tissues		
	Number positive (%)	Number negative (%)	
Serum HBsAg			
Positive	12 (66.7)	6 (33.3)	*p* = 0.007
Negative	2 (16.7)	10 (83.3)	

(B)	HBV cccDNA in HCC tissues		
	Number positive (%)	Number negative (%)	
Serum HBsAg			
Positive	1 (5.6)	17 (94.4)	*p* = 1.0
Negative	0 (0)	12 (100)	

### Correlation between intrahepatic HBsAg and intrahepatic HBV cccDNA

**Fig. 3:**
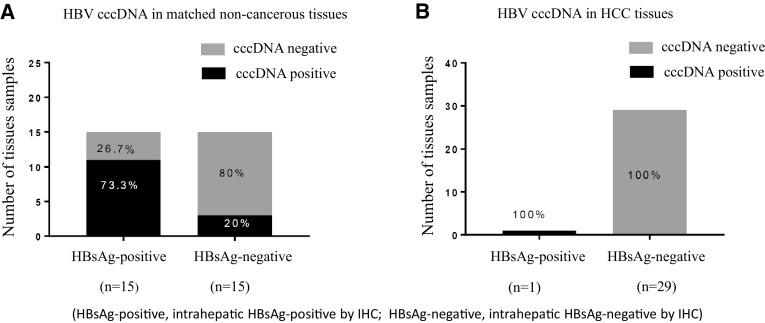
Correlation between intrahepatic HBsAg and intrahepatic HBV cccDNA in matched non-cancerous tissues (**a**) and HCC tissues (**b**)

**Fig. 4:**
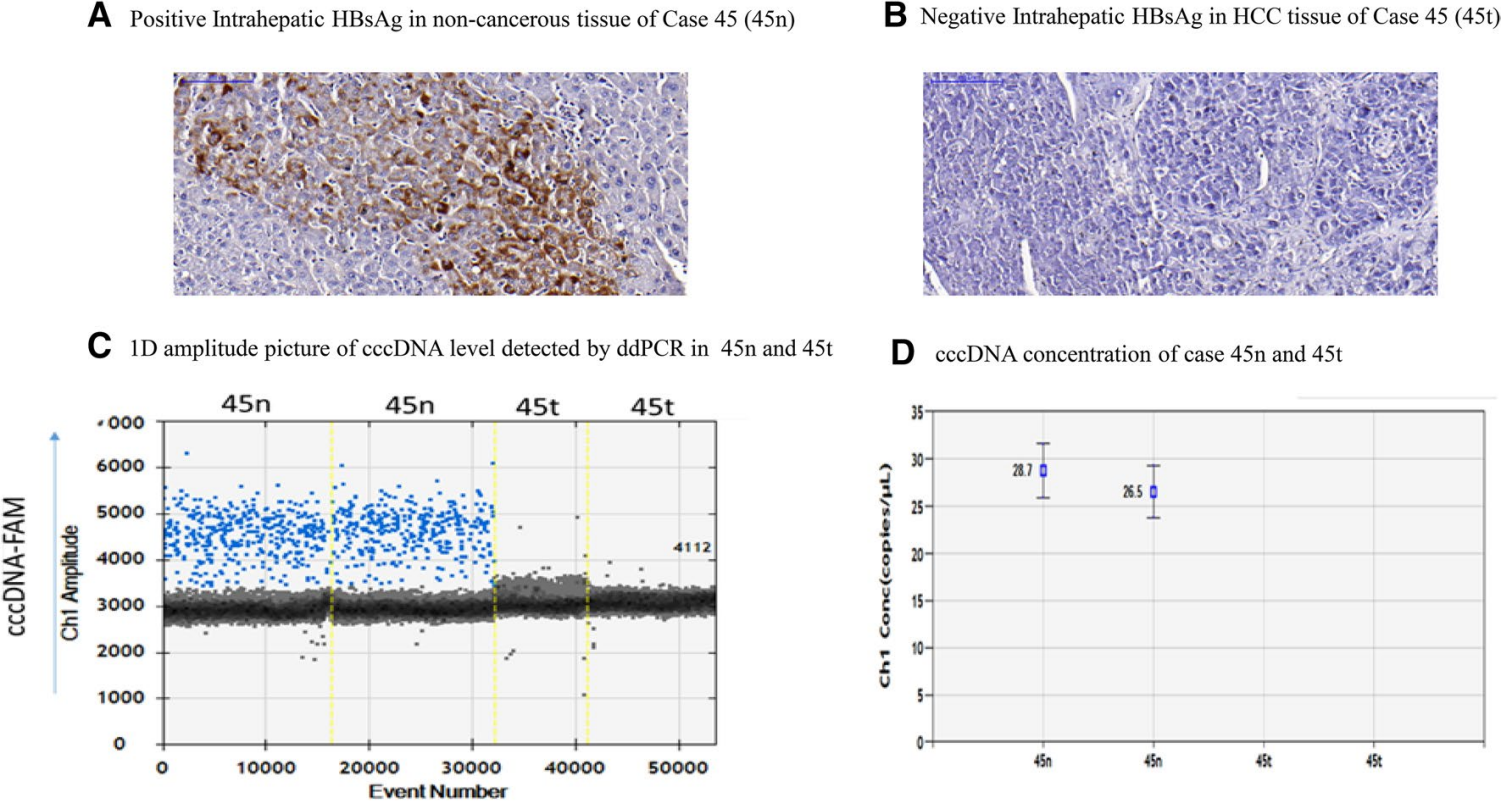
Representative of positive intrahepatic HBsAg in non-cancerous tissue (**a**), and negative intrahepatic HBsAg in HCC tissue (**b**) evaluated by IHC. Intrahepatic cccDNA quantitated by ddPCR revealed positive in matched non-cancerous tissue but negative in HCC tissue of case 45 (**c**, **d**)

Both intrahepatic HBsAg and intrahepatic cccDNA were evaluated in 30 pairs of matched non-cancerous and HCC tissues. The clinicopathological features are illustrated in Table 3. In 15 matched non-cancerous tissues with positive staining for intrahepatic HBsAg, intrahepatic cccDNA was detected in 73.3% (11/15); in 15 matched non-cancerous tissues with negative staining for intrahepatic HBsAg, intrahepatic cccDNA was detected only in 20% (3/15), as shown in Table 5a. Interestingly, in HCC tissues, both intrahepatic HBsAg and intrahepatic cccDNA were detected only in the same 1 HCC tissue (2 copies/ng DNA) but not in the other 29 HCC tissues, as shown in Table 5b and Fig. 3. Representative of intrahepatic HBsAg and cccDNA is shown in Fig. 4. Significant correlations between intrahepatic HBsAg and cccDNA were found in both matched non-cancerous (*p* < 0.01) and HCC tissues (*p* < 0.05).

**Table 5:** Correlation between intrahepatic HBsAg and intrahepatic HBV cccDNA (n = 30) in matched non-cancerous tissues (A) and in HCC tissues (B)

(A)	HBV cccDNA in matched non-cancerous tissues		
	Number positive (%)	Number negative (%)	
Intrahepatic HBsAg			
Positive	11 (73.3)	4 (26.7)	
Negative	3 (20.0)	12 (80.0)	*p* = 0.003

(B)	HBV cccDNA in HCC tissues		
	Number positive (%)	Number negative (%)	
Intrahepatic HBsAg			
Positive	1 (100)	0 (0)	
Negative	0 (0)	29 (100)	*p* = 0.033

## Discussion

Serum HBsAg is an important diagnostic marker for HBV infection. However, some studies suggest that HBV DNA can be detected in the liver tissue of HBsAg seroclearance or patients with occult HBV, mainly in the form of cccDNA, and these patients can continue to develop HCC [16, 17]. In the present study, we investigated intrahepatic HBsAg in both matched non-cancerous and HCC tissues by IHC, and found that serum HBsAg was significantly associated with intrahepatic HBsAg only in matched non-cancerous tissues. In contrast to matched non-cancerous tissues, no association between serum HBsAg and intrahepatic HBsAg in HCC tissues was observed. In serum HBsAg-positive group, positive intrahepatic HBsAg was detected in matched non-cancerous and HCC tissues at the frequency of 73.2 and 10.7%, respectively. Our results showed that intrahepatic HBsAg was reduced in most HCC tissues, which is consistent with the finding of Tian et al. [18], Jing et al. [19] and Wang [20], but the exact mechanism is still unclear. The finding that intrahepatic HBsAg could not be detected in HCC tissues by IHC from patients with positive serum HBsAg might be explained as follows, (1) HBV in HCC tissues could not express HBsAg, (2) HBsAg in HCC tissues could not be detected by the antibody used in this study, (3) HBV DNA is no longer present in HCC tissues. Therefore, in this study, so as to explore whether the decreased expression of HBsAg in HCC tissues is caused by HBV cccDNA reduction, intrahepatic HBV cccDNA was quantitated by ddPCR in both negative intrahepatic HBsAg and positive intrahepatic HBsAg tissues.

In the present study, the significant correlation between serum HBsAg and intrahepatic cccDNA was found only in matched non-cancerous tissues. This is different from previous studies; as Fu et al. reported that there was no correlation between serum HBsAg and intrahepatic cccDNA in HBV-related hepatocellular carcinoma [11], but Wang et al. [13] reported correlation between serum HBsAg and intrahepatic cccDNA in both tumor and non-neoplastic liver tissues of HBV-associated HCC patients. The reasons for variety of results are inconclusive. But the possible contributing factor might be the size of tumors in tissue samples, in which 60% of specimen in this study was larger than 5 cm. Recently, Wang et al. [10] reported that cccDNA copy number per tumor in early tumors (smaller than median, ≤ 3 cm) was higher than those in larger tumors (> 3 cm); furthermore, in patients with small tumors, the cccDNA copy number did not differ between tumor and paired non-neoplastic liver, whereas in patients with larger tumors, the cccDNA copy in tumor was significantly lower than that of the paired non-neoplastic liver. Another striking finding of the present study was the significant correlation between intrahepatic HBsAg and intrahepatic cccDNA in both matched non-cancerous and HCC tissues. Furthermore, a significant reduction of intrahepatic cccDNA in HCC tissues was observed, which is consistent with two previous studies [6, 7] but not consistent with some other studies [8, 11, 12]. The discordance of the research results might be due to the tissue sampling. Of 15 tissues with negative intrahepatic HBsAg, intrahepatic cccDNA could be detected in 3 (20%) tissues. On the other hand, of 15 tissues with positive intrahepatic HBsAg, intrahepatic cccDNA could not be detected in 4 (26.7%) tissues. These discordant findings might be due to heterogeneity of HBV infection in tissues, and tissue sampling for IHC and ddPCR might not be from the same area.

Our finding demonstrated the followings: (1) In matched non-cancerous tissues, serum HBsAg significantly correlated with both intrahepatic HBsAg and cccDNA; (2) In HCC tissues, serum HBsAg did not correlate with both intrahepatic HBsAg and cccDNA; (3) Intrahepatic HBsAg significantly correlated with intrahepatic cccDNA in both matched non-cancerous and HCC tissues; (4) The intrahepatic cccDNA level in HCC tissues was significantly lower than that in matched non-cancerous tissues. We concluded that intrahepatic HBsAg reduction in HCC tissues was correlated with HBV cccDNA reduction. The reduction of cccDNA may be due to factors controlling proliferation of cancerous cells and/or factors inducing eradication of intrahepatic cccDNA, which lead to reduced expression of HBsAg, including other HBV viral antigens, and consequently help cancerous cells avoid immune recognition and destruction. Therefore, factors that are involved in cccDNA reduction of HBV-associated HCC tissues might contribute to HCC progression and immune evasion. Our findings warrant further investigation into the cause(s) of cccDNA reduction in HBV-associated HCC tissues, which might yield immune-related therapy for HBV-associated HCC.
